# Clinical implementation of a cervical cancer screening program via co-testing at a university hospital

**DOI:** 10.1371/journal.pone.0278476

**Published:** 2022-12-01

**Authors:** Valeria Denninghoff, Felicitas von Petery, Cristóbal Fresno, Mercedes Galarza, Florencia Torres, Alejandra Avagnina, Vanina Fishkel, Hugo Krupitzki, Angel Fiorillo, Fernando Monge

**Affiliations:** 1 Molecular-Clinical Laboratory, University of Buenos Aires—National Council for Scientific and Technical Research (CONICET), Buenos Aires, Argentina; 2 Center for Medical Education and Clinical Research (CEMIC), Buenos Aires, Argentina; 3 Health and Sciences Research Center (CICSA), Health and Sciences Faculty, Anahuac University, Huixquilucan, Estado de México, México; 4 General Acute’s Hospital Dr. I. Pirovano, Buenos Aires, Argentina; 5 CEMIC-CONICET Interacting Units, Buenos Aires, Argentina; Kasturba Medical College Mangalore, Manipal Academy of Higher Education, INDIA

## Abstract

The Human Papillomavirus (HPV) test is a crucial technology for cervical cancer prevention because it enables programs to identify women with high-risk HPV infection who are at risk of developing cervical cancer. Current U.S. Preventive Services Task Force recommendations include cervical cancer screening every three years with cervical cytology alone or every five years with either high-risk HPV testing alone or high-risk HPV testing combined with cytology (co-testing). In Argentina, 7,548 new cervical cancer cases are diagnosed each year with 3,932 deaths attributed to this cause. Our study aims to show the clinical implementation of a cervical cancer screening program by concurrent HPV testing and cervical cytology (co-testing); and to evaluate the possible cervical cancer screening scenarios for Latin America, focusing on their performance and average cost. A cervical cancer screening five year program via co-testing algorithm (Hybrid-2-Capture/cytology) was performed on women aged 30–65 years old at a university hospital. Statistical analysis included a multinomial logistic regression, and two cancer screening classification alternatives were tested (cytology-reflex and HPV-reflex). A total of 2,273 women were included, 91.11% of the participants were double-negative, 2.55% double-positive, 5.90% positive-Hybrid-2-Capture-/negative-cytology, and 0.44% negative-Hybrid-2-Capture/positive-cytology. A thorough follow-up was performed in the positive-Hybrid-2-Capture group. Despite our efforts, 21 (10.93%) were lost, mainly because of changes on their health insurance coverage which excluded them from our screening algorithm. Of the 171 women with positive-Hybrid-2-Capture results and follow-up, 68 (39.77%) cleared the virus infection, 64 (37.43%) showed viral persistence, and 39 (22.81%) were adequately treated after detection via colposcopy/biopsy of histological HSIL (High-Grade Squamous Intraepithelial Lesion). The prevalence of high-risk HPV in this population was 192 women (8.45%), with HSIL histology detection rates of 17 per 1,000 screened women. A multinomial logistic regression analysis was performed over the women with positive-Hybrid-2-Capture considering the follow up (clearance, persistence and HSIL) as dependent variable, and the cytology test results (positive- or negative-cytology and Atypical Squamous Cells of Undetermined Significance, ASC-US) as independent variable. The model supported a direct association between cytology test results and follow up: negative-cytology/clearance, ASC-US/persistence, and positive-cytology/HSIL with the following probabilities of occurrence for these pairs 0.5, 0.647 and 0.647, respectively. Cytology could be considered a prognostic-factor in women with a positive-Hybrid-2-Capture. These findings suggest that the introduction of co-testing could diminish the burden of cervical cancer in low-and middle-income-countries, acting as a tool against inequity in healthcare.

## Introduction

**Fig 1:**
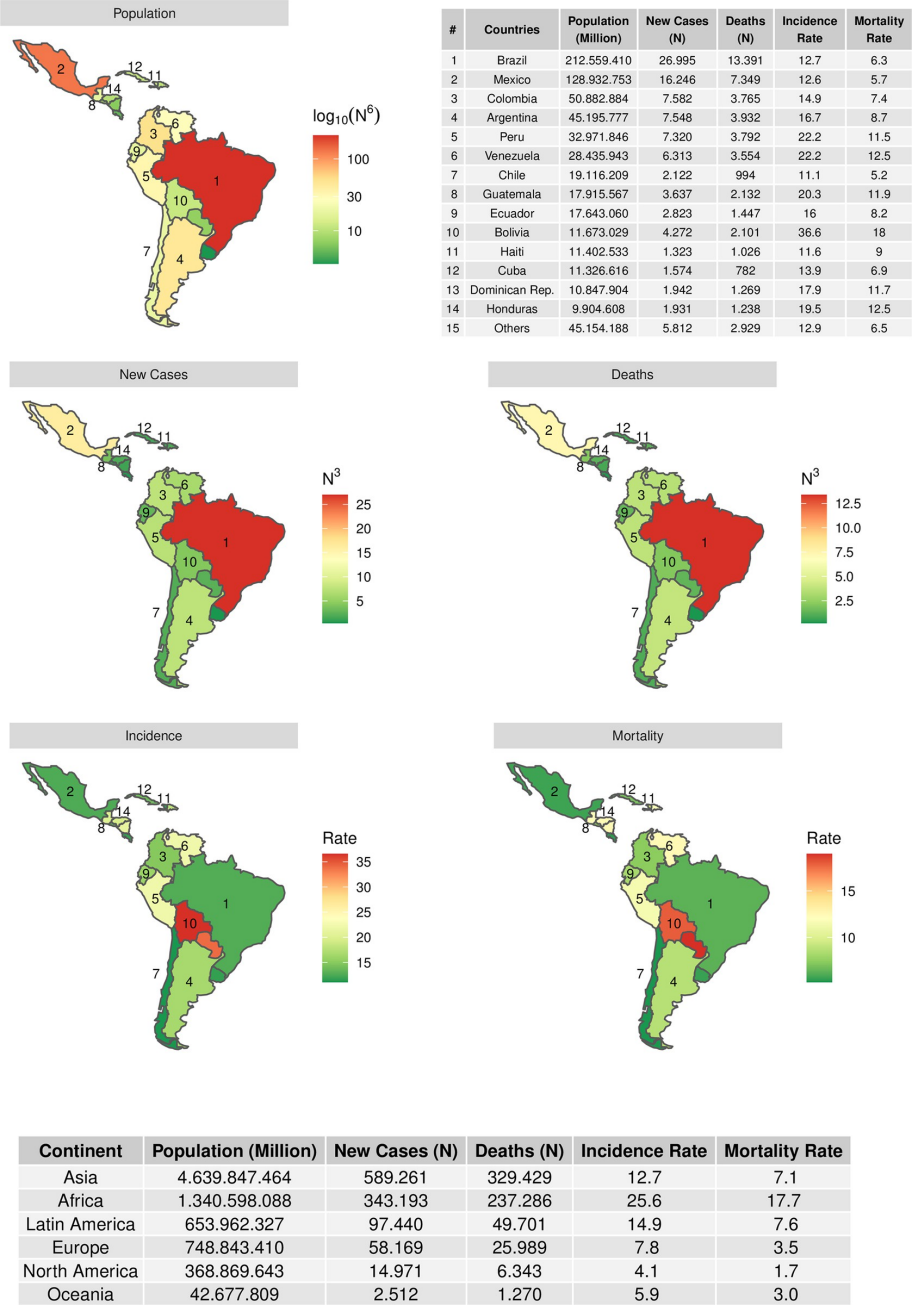
Estimated incidence and mortality rates in 2020 in females of all ages for the six continents, and the top 20 Latin America and the Caribbean. Data obtained from [1].

In 2020, cervical cancer was the fourth most common cancer in women globally, with an incidence of 13%, resulting in 341,831 deaths worldwide, over 90% of these occurring in low- and middle-income countries (LMIC). Almost 86% of cases and 88% of deaths occur in developing countries, resulting in the third cause of death among women in Latin America [1]. Also, in 2020, COVID-19 exposed Latin America’s inequality, where three-quarters of Latin Americans are of low- or lower-middle income, and only 3% are classified as high income. Latin America has 653 million inhabitants, distributed mainly in Brazil (33%), Mexico (20%), Colombia (8%), Argentina (7%), and the remaining 32% in the other 14 countries (Fig 1). Regardless of population and distribution, they all share the same common problems: accessibility, inequality, segmentation, and poverty; 82–90% of the population is located in urban centers, with real-time cancer burden strongly determined by socioeconomic characteristics and geographical barriers [2].

The International Agency for Research on Cancer (IARC) has classified 12 Human Papillomavirus (HPV) types as group 1 carcinogens considering them pathogenic or high-risk HPV due to their link to the development of cervical cancer [3–5]. Although most sexually active females become infected with high-risk HPV (hrHPV) types once in their lifetime, less than 10% of women become persistently infected, a condition required for cervical cancer development [6–8]. The HPV test is a crucial technology for cervical cancer prevention because it enables programs to identify women with high-risk HPV infection. Current U.S. Preventive Services Task Force recommendations include cervical cancer screening every three years with cervical cytology alone or every five years with either high-risk HPV testing alone or high-risk HPV testing combined with cytology (co-testing) [9, 10].

Although it can be almost entirely prevented, cervical cancer remains a significant public health problem in Argentina, with 7,548 new cases diagnosed yearly and 3,932 deaths attributed to this cause [1]. In addition to race/ethnicity and geography, health insurance coverage is essential in access to cervical cancer screening. In most countries, healthcare is polarized in public and private. Our population has access to health divided into three subgroups: private (pay to access), public (access provided by the state), and rural (limited or no access due to geographical barriers). Based on this evidence, in 2011, the Argentine National Cancer Institute and the National Program for the Prevention of Uterine Cervical Cancer incorporated hrHPV testing as a tool for cervical cancer screening, starting in the Province of Jujuy and later extending to other provinces [11]. The program was implemented between 2012 and 2014; women from the remaining 22 regions had no access to this technology at this time, thus revealing that the health program described cannot yet be considered a national public health intervention. In this context, our study aims to show the implementation of a cervical cancer screening program by concurrent HPV testing and cervical cytology (co-testing), in women aged 30–65 years, at a University Hospital setting (private health group) with strict stringency; and to analyze the five-year cumulative incidence rate of cervical cancer. Secondary outcomes include (a) determining the prevalence of hrHPV in this population; (b) describing the study population during follow-up (maximum interval of five years), focusing on the diagnosis of high-grade lesions and cervical cancer, persistence, and clearance of viral infection; and (c) to evaluate the possible cervical cancer screening scenarios for Latin America, focusing on their performance and average cost.

## Methods

### Cohort description

**Fig 2:**
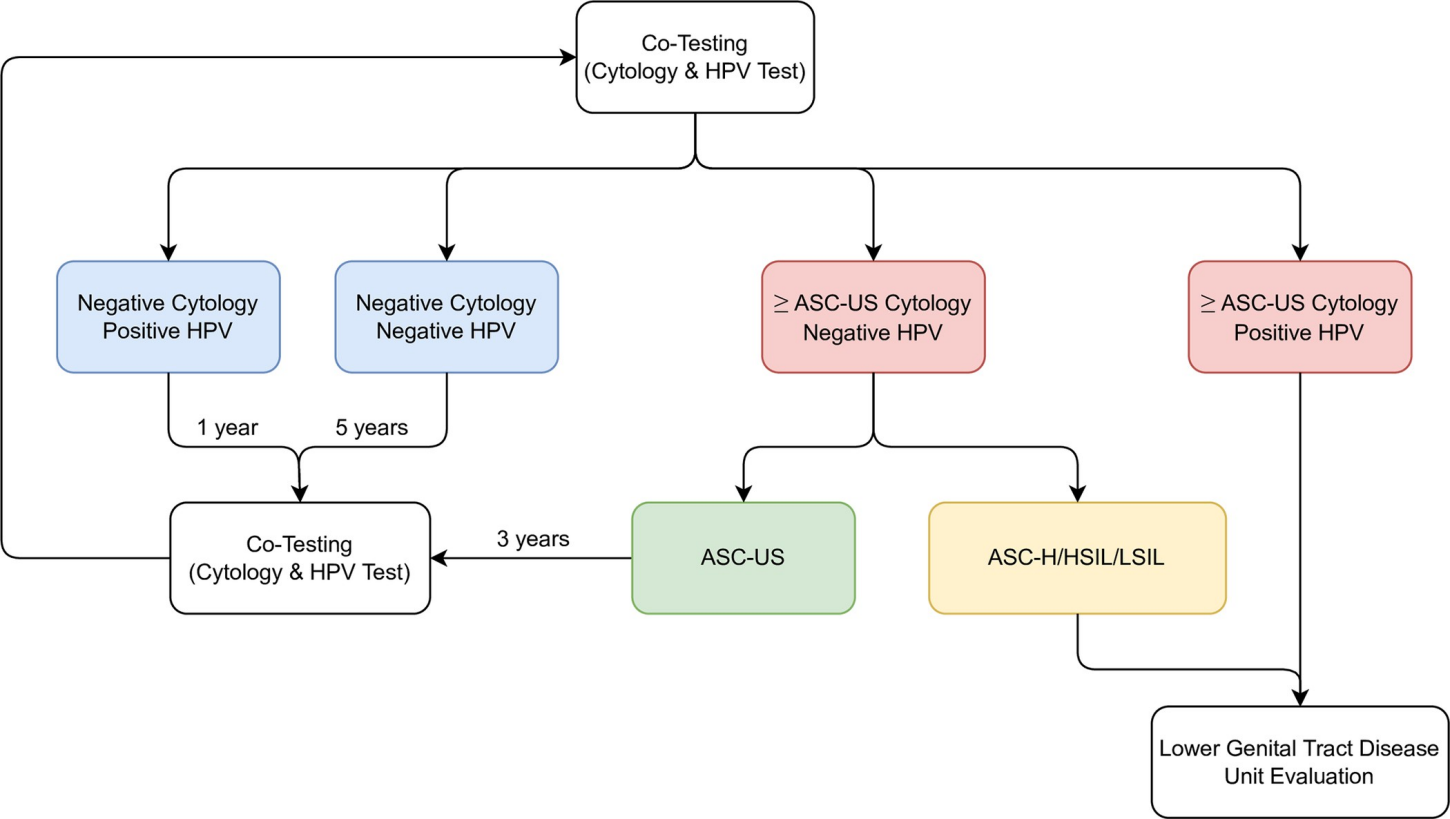
Cervical cancer screening algorithm via co-testing. The molecular test has an international cost of USD$250. The evaluations that include the clinical time and reagents are equivalent to 12% of the value: Lower Genital Tract Disease Unit Evaluation USD$4 (2%), Cytology USD$4 (2%), Colposcopy USD$2 (0.8%), Biopsy USD$10 (4%), Tissue processing and analysis USD$11 (4%). The costs of "clinical interventions" were not considered because, in our country, they are negligible in comparison to molecular methods. Code used: Human Papillomavirus (HPV); Atypical squamous cells, which cannot exclude a high-grade squamous intraepithelial lesion (ASC-H); Atypical Squamous Cells of Undetermined Significance (ASC-US); High-Grade Squamous Intraepithelial Lesion (HSIL); Low-Grade Squamous Intraepithelial Lesion (LSIL); Atypical Glandular Cells (AGC); Adenocarcinoma In Situ (AIS).

Non-pregnant women between the ages of 30 and 65 with no prior history of high-grade cervical lesions (reported in the last 20 years of their lives) and no clinical findings of any HPV related condition at the time of screening, who usually seek medical attention at the hospital, were enrolled prospectively between July 2013 and June 2018 at a University Hospital (Fig 2). This protocol was reviewed and approved by the Center for Medical Education and Clinical Research (CEMIC) Institutional Review Board (IRB #837–2013). After a detailed explanation regarding the study, participants who agreed to sign a written consent were recruited (Form #474–2013). The trial was designed to perform a single 5-year cycle to evaluate the methodology and its application. Samples were obtained by clinicians adopting a parallel/double-blind strategy. After completing the 5-year period, no new data was included. The study was conducted under the principles of Good Clinical Practice Guidelines and the Helsinki Declaration. The implementation of this program was possible thanks to the inclusion of co-testing into CEMIC’s private health insurance coverage.

### Data acquisition

The conventional Papanicolaou smear samples were stained and examined under an optical microscope. It is a multichromatic stain that uses five dyes to stain various components of the cells differentially: hematoxylin; Orange G; Eosin Y; Light Green SF yellowish; and Bismarck brown Y, in 95% ethyl alcohol. The stain should result in fairly transparent cells so we can interpret even thicker specimens with overlapping cells [12]. Colposcopy is a method of examination of the cervix using an optical magnifying instrument [13].

The cervical samples for viral determination (Digene HPV HC2 DNA Test®) were taken with Digene Cervical Sampler®, placed in a transport medium, preserved adequately, and processed using the Hybrid-2-Capture technique (Quiagen®, USA). The cutoff value in the Relative Light Unit/cutoff ratio (RLU/CO) was ≥1 pg/mL for 13 high-risk HPV types. Hybrid-2-Capture technology detected RNA:DNA hybrids using signal-amplified, chemiluminescent technology, with full genome probes complementary to HPV DNA. The resulting signal was automatically interpreted using the DML instrument with the Digene Hybrid Capture System Version 2 Software. According to the co-test results and following the screening algorithm (Fig 2), women were retested at set intervals or referred to the Lower Genital Tract Disease Unit for colposcopic evaluation. Biopsies were performed when necessary. Women with histologic lesions were managed following the 2012 Updated Consensus Guidelines [14].

### Statistical analysis

Cohort descriptive statistical analysis over the age and turnaround time included variable range, mean and standard deviation over decade groups (30s, 40s, 50s and 60s). Analysis of variance (ANOVA) test was performed over turnaround time group means. Posterior Fisher’s Least Significant Difference test was performed with Bonferroni p-value correction for multiple comparisons. Homogeneity of variances was tested with Bartlett’s test. Co-test results were presented as contingency tables over the decade groups, and analyzed with *χ^2^* test. Companion alluvial plot for data distribution was also created, to get a graphical cohort distribution picture. Multinomial logistic regression was used to assess the cytology impact over the follow-up outcome for clearance or histologic HSIL (HSIL-h) compared to the persistence counterpart. Finally, classification performance statistics were calculated for the proposed screening strategies: HPV-cytology (co-testing), serialized HPV-cytology, and serialized cytology-HPV testing. The performance measurements included: sensitivity, specificity, positive/negative predictive value, positive/negative likelihood ratio and diagnostic odds ratio. The diagnostic odds ratio will be the key metric to test the effectiveness of screening strategies proposed in this manuscript as diagnostic tests. It is calculated as the positive likelihood ratio divided by the negative likelihood ratio. As such it is an odds ratio, where the greater the number the better the diagnostic test, from a medical perspective. All statistical tests, except where noted, were two-sided with a statistical significance *α = 0.05* and were performed using R version 4.0.5. Complete cohort database can be found in S1 File.

## Results

### Cohort screening

A total of 2,273 patients (2,386 samples) met the eligibility criteria and were included in the study, with a mean age ± SD of 44.95±9.84 years old, and a mean turnaround time ± SD of 19.31±9.34 days. Independent analysis of the two methods showed that 192 women (8.45%) had positive-Hybrid-2-Capture (HC2); and 2,205 (97.01%), 28 (1.23%) and 40 (1.76%) women had negative, Atypical Squamous Cells of Undetermined Significance (ASC-US), and positive-cytology results respectively. For the data analysis, we decided to separate the ASC-US cytology because of the varying risk associated with this particular cytology according to age and high-risk HPV status [15]. The study cohort data for screening via co-testing are shown in Table 1; the analysis was performed according to age groups.

**Table 1:** Study cohort descriptive data.

Variables		Cohort Groups				
		30s	40s	50s	60s	Total
Subjects	N (Percentage)	858 (37.75%)	749 (32.95%)	468 (20.59%)	198 (8.71%)	2273 (100%)
Age [years]	Range (min-max)	30–40	41–50	51–60	61–65	30–65
	Mean±SD	34.88±3.34	45.36±2.88	55.15±2.93	62.91±1.42	44.95±9.84
HC2 (pos|neg) [subjects] P<0.001	ASC-US	13|4	3|2	0|2	4|0	20|8
	Negative	73|741	37|699	17|445	7|186	134|2071
	Positive	25|2	8|0	4|0	1|0	38|2
	Total	111|747	48|701	21|447	12|186	192|2081
Follow-up for pos-HC2 (ASC-US|neg|pos) [subjects] N = 192 (8.45%) P<0.001	Subjects [N] (Percentage)	111 (57.81%)	48 (25%)	21 (10.94%)	12 (6.25%)	192 (100%)
	Lost	2|9|2	1|3|1	0|1|1	0|1|0	3|14|4
	Clearance	3|29|1	0|20|3	0|8|1	0|3|0	3|60|5
	Persistence	5|23|4	2|12|2	0|8|1	4|3|0	11|46|7
	HSIL-h	3|12|18	0|2|2	0|0|1	0|0|1	3|14|22
	Total	13|73|25	3|37|8	0|17|4	4|7|1	20|134|38
Discordant Data (pos-Cytology|neg-HC2) [subjects] N = 10 (0.44%)	CIN 1	1	0	0	0	1
	ASC-H	1^a^	0	0	0	1
	ASC-US	4	2	2	0	8
	Total	6	2	2	0	10
	(Percentage)	(60%)	(20%)	(20%)	(0%)	(100%)

Cytology test results positive (pos) or negative (neg); Hybrid-2-Capture (HC2); High-Grade Squamous Intraepithelial Lesion (HSIL); histologic HSIL (HSIL-h); Cervical Intraepithelial Neoplasia (CIN); Atypical squamous cells, which cannot exclude a high-grade squamous intraepithelial lesion (ASC-H); Atypical Squamous Cells of Undetermined Significance (ASC-US); SD: Standard Deviation. Model results included two p-values for time between sample collection and process variables. The first one corresponds to group means using analysis of variance (ANOVA), where the second one is Bartlett’s test of homogeneity of variances. Categorical contingency tables were analyzed with a *χ^2^* test for the complete data.

^a^ Revision of cytology informed negative-cytology no abnormal coloscopic findings. Patient rescreened at three months tested double negative.

### Parallel testing (co-testing) results

**Fig 3:**
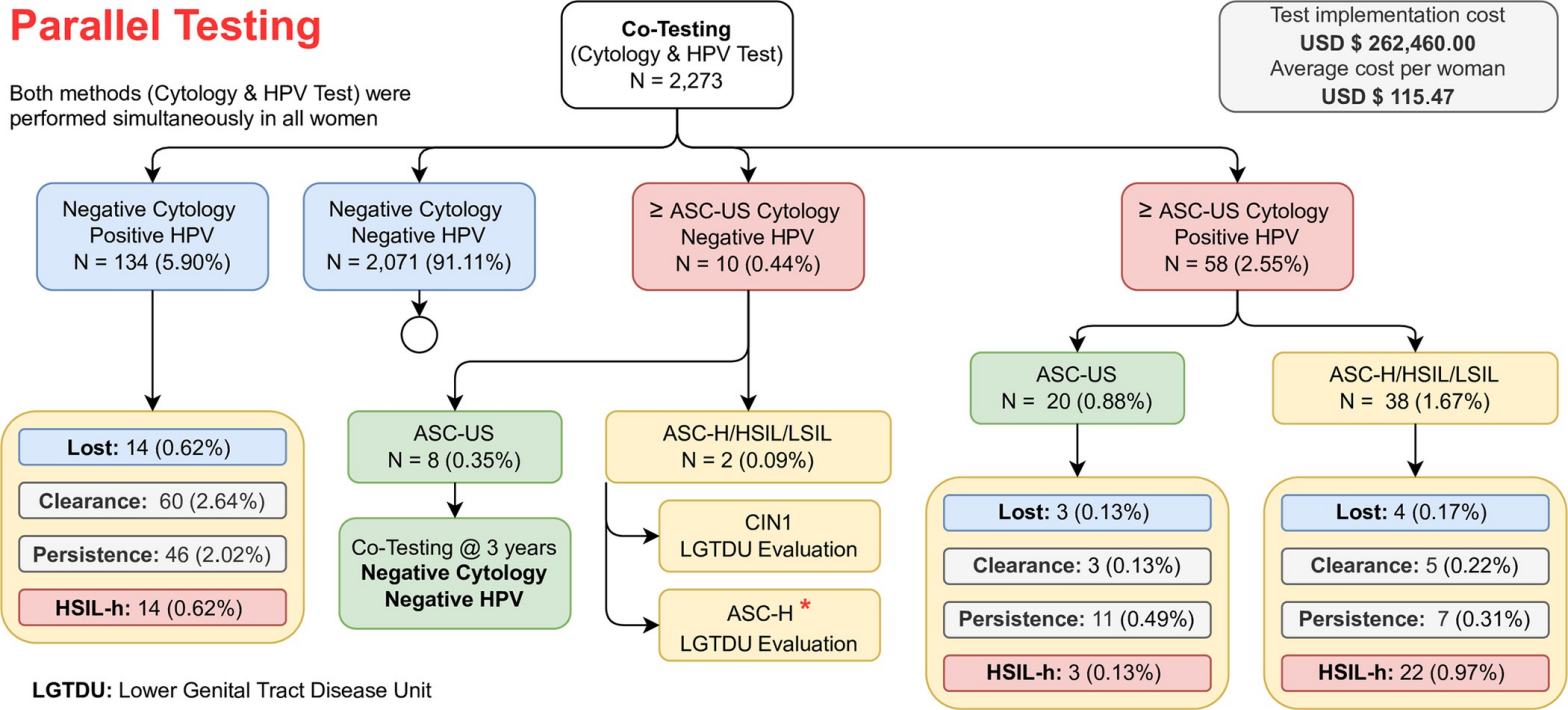
Results of the cervical cancer screening via co-testing algorithm. Atypical Squamous Cells of Undetermined Significance (ASC-US); Cervical Intraepithelial Neoplasia (CIN); Atypical squamous cells (ASC); Human Papillomavirus (HPV); Atypical Glandular Cells (AGC); Adenocarcinoma In Situ (AIS).

Fig 3 enables the analysis of data obtained after study population screening performed with both tests combined (co-testing), double-blind, and parallel. These patients were clinically followed for the duration of the screening program. We observed discordant data in 10 cases: eight ASC-US and two >ASC-US cytology (0.09%). In the Cervical Intraepithelial Neoplasia (CIN)1/negative-Hybrid-2-Capture patient, no abnormal colposcopic findings were observed (adequate colposcopy, Transformation Zone type 1) [16]. She was rescreened at six months, testing negative for both methods. In the Atypical squamous cells, which cannot exclude a high-grade squamous intraepithelial lesion (ASC-H)/negative-Hybrid-2-Capture patient, no abnormal colposcopic findings were observed (adequate colposcopy, Transformation Zone type 1) [16], cytology was revised and reclassified as negative. This patient was rescreened at three months, testing negative for both methods.

### Population follow-up

**Fig 4:**
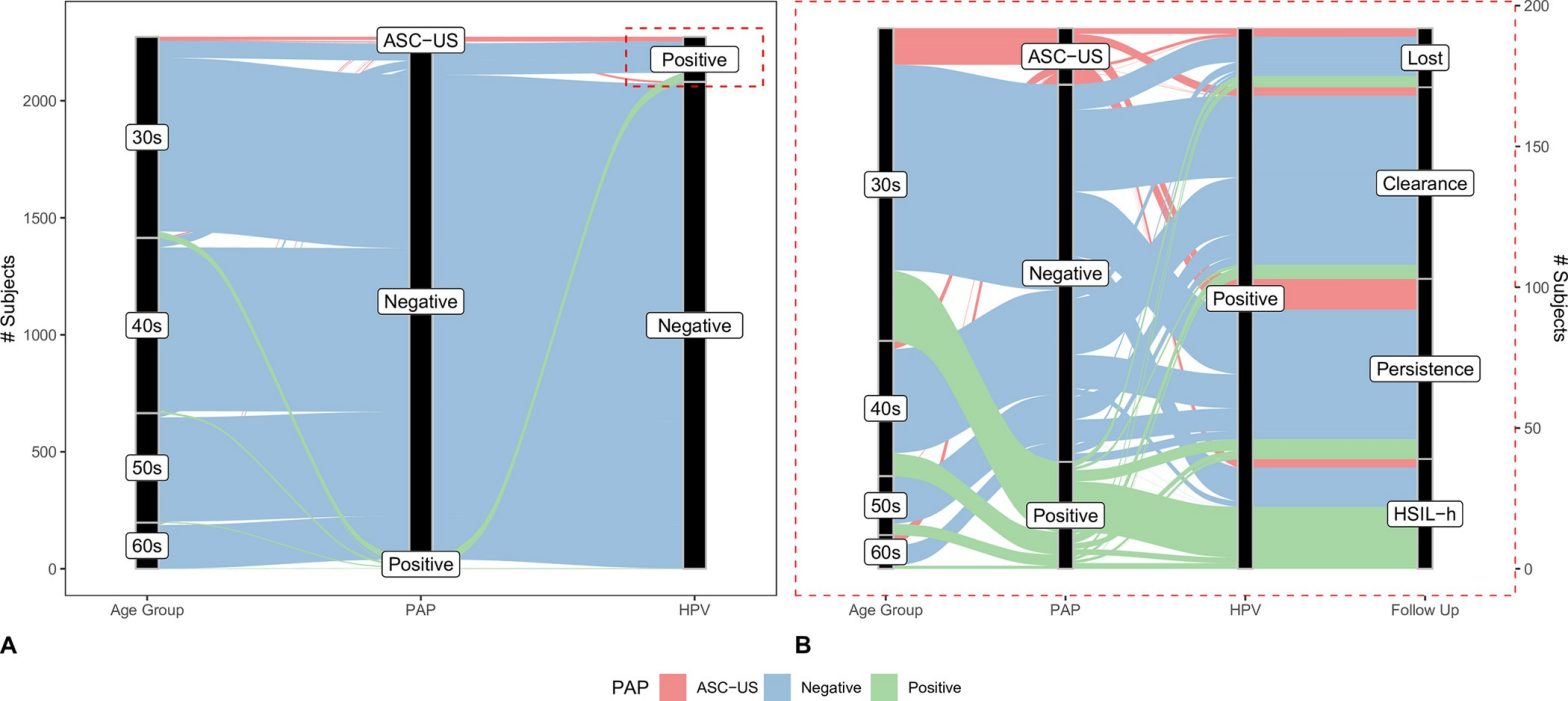
Categorical cohort plot. Cytology (PAP); High-Grade Squamous Intraepithelial Lesion (HSIL) histological-HSIL (HSIL-h). A) Alluvial plot representation of 2,273 patients (lines), where there are three categorical groups in the x-axis (age group, cytology and Human Papillomavirus (HPV)-test results). Within the age group, the subjects are clustered according to the decade strata (the 30s, 40s, 50s or 60s), whereas cytology results use Atypical Squamous Cells of Undetermined Significance (ASC-US), negative or positive strata, and HPV only positive or negative. The Alluvial span, i.e., horizontal x-axis splines are filled by ASC-US, negative and positive cytology results with red, blue, and green, respectively. The vertical axis depicts the cumulative number of subjects. Interestingly, almost all the subjects, regardless of the decade they belonged to, had a negative cytology result with its corresponding negative HPV counterpart in most of the cases. B) The HPV-positive subjects zoom-in (dashed red rectangle in the left panel) with 192 patients. An additional categorical axis includes subject’s follow-up (lost, clearance, persistence or HSIL-h). For easy visualization, the alluvial spans are filled using the follow-up categories. An auxiliary right y-axis was included according to the number of subjects in the zoom-in panel.

In order to get a clear picture of the five-year patient follow-up, we constructed an alluvial plot (Fig 4). Each line represents a patient, and its color is related to the cytology result. A thorough follow-up was performed in the positive-Hybrid-2-Capture group. Despite our efforts, 21 (10.93%) were lost, mainly because of changes in their health insurance coverage which excluded them from our screening algorithm. Of the 171 women with positive-Hybrid-2-Capture results and follow-up, 68 (39.77%) cleared the virus infection, 64 (37.43%) showed viral persistence, and 39 (22.81%) were adequately treated after detection via colposcopy/biopsy of histological HSIL. Regarding effectiveness, the HSIL histology detection rate was 17 per 1,000 screened women.

### Cytology direct association with five-year follow-up

**Fig 5:**
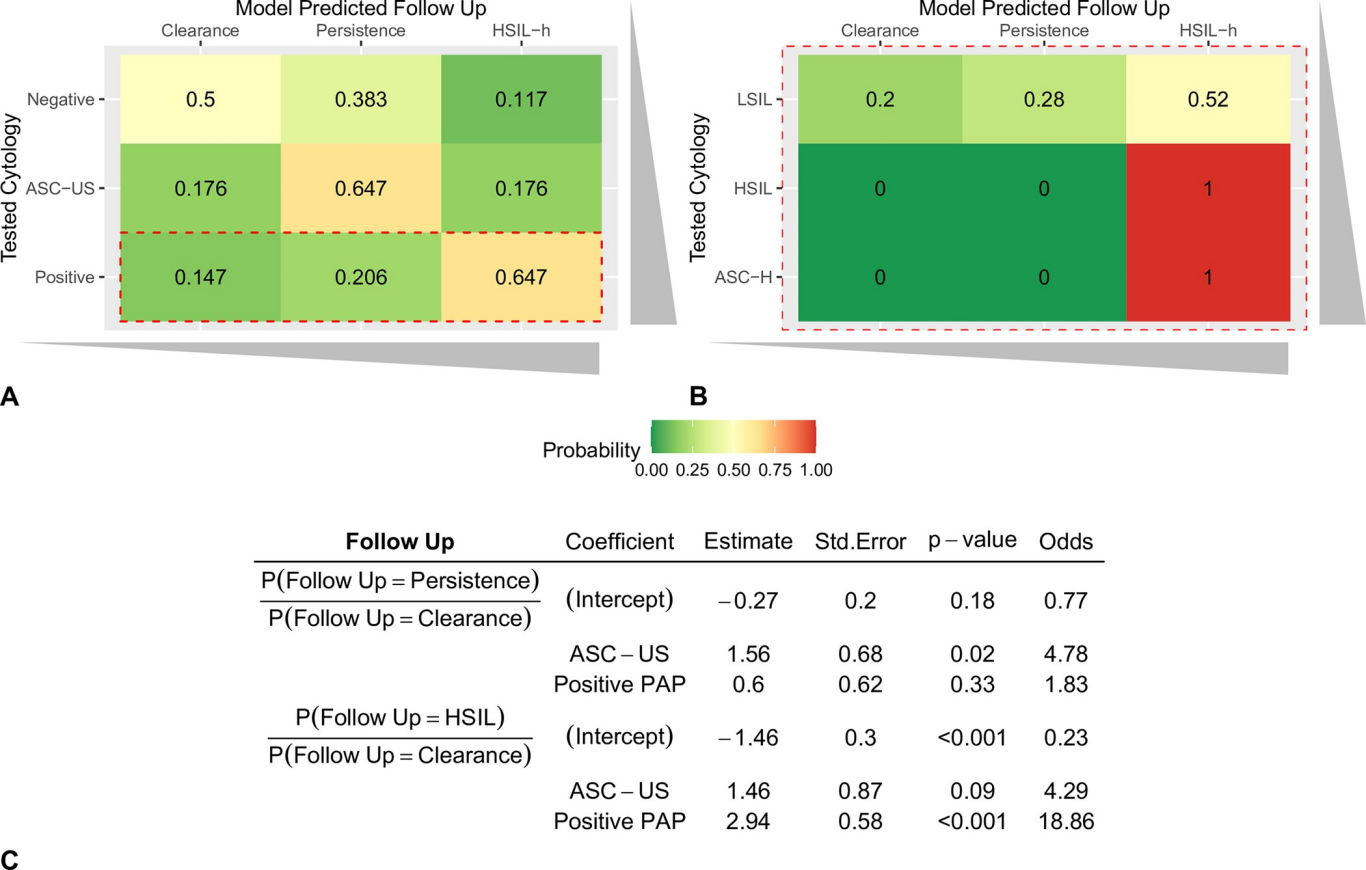
Multinomial logistic regression results for the five-year follow-up analysis of the 171 women with positive-Hybrid-2-Capture results. Interestingly, model predicted follow-up probability could be associated with a preponderant cytology test result. A) Negative, Atypical Squamous Cells of Undetermined Significance (ASC-US), or positive-cytology test results to predict associated follow-up class probability. These results support a direct association with prognosis, i.e., negative-cytology/clearance (P = 0.5), ASC-US/persistence (P = 0.647, 4.78 odds ratio), and positive-cytology/High-Grade Squamous Intraepithelial Lesion (HSIL) (P = 0.647, 19.09 = 18.86 + 0.23 odds ratio). B) Zoom-in for positive-cytology into Low-grade squamous intraepithelial lesion (LSIL), HSIL or Atypical squamous cells, which cannot exclude high-grade squamous intraepithelial lesion (ASC-H) results. It is worth noting that for more aggressive positive cytology, such as HSIL or ASC-H, the predicted follow-up has no discrepancy for histological-HSIL (P = 1). C) Multinomial logistic regression model parameters, where the clearance follow-up class has been chosen as reference to compare against persistence or HSIL cases.

To explore the existence of cytology test results with the five-year follow-up we constructed a multinomial logistic regression model with 171 women with positive-Hybrid-2-Capture results (Fig 5). The model predicted that follow-up outcome probability could be associated with a predominant cytology result, i.e., negative-cytology/clearance (P = 0.5), ASC-US/persistence (P = 0.647, 4.78 odds ratio), and positive-cytology/HSIL (P = 0.647, 19.09 total odds ratio).

### Estimated accuracy of cytology, Hybrid-2-Capture and co-testing

**Fig 6:**
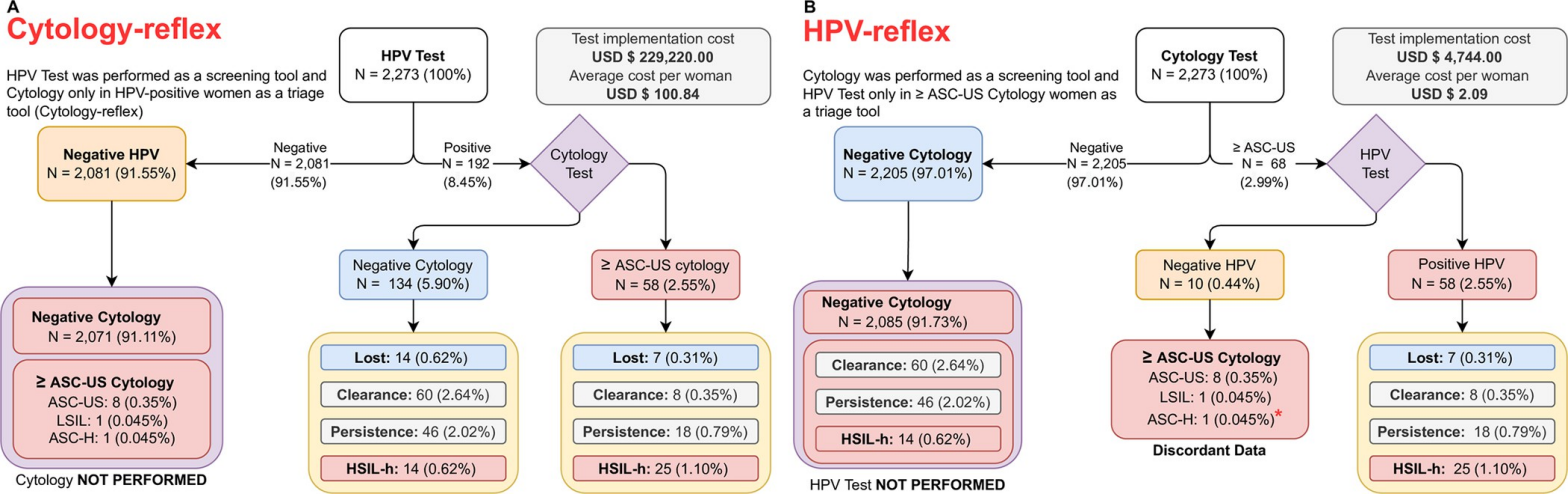
Cervical cancer screening via reflex algorithm. A) cytology-reflex B) Human Papillomavirus (HPV)-reflex. Clinician time, reagent, and pathological test equivalent to 12% of the molecular methods (USD$250), thus the costs of "clinical interventions" were not considered because, in our country, they are negligible in comparison to molecular tests.

In order to assess the classification performance of the proposed screening strategies for cervical cancer, we had to transform the original results obtained from Fig 2 outlines the data into different scenarios constructing 2-by-2 contingency tables to obtain the related metrics (sensitivity, specificity, positive/negative predictive value, positive/negative likelihood ratio and diagnostic odds ratio). We evaluated both tests (HC2 and cytology) vs. follow-up (histological-HSIL as the primary end-point) in a) both tests independently and in b) combined (co-testing). Since the analysis is binomial, the following scenarios were proposed adding discordant co-testing to negative or positive binary values: b1) negative co-test = double-negative and discordant, and b2) positive co-test = double-positive and discordant. In the b2 study, we had to explain the positive co-test = double-positive and discordant cases (Table 2). Interestingly, b2 co-testing strategy outperforms b1 counterpart with a diagnostic odds ratio of 1,148 versus 145. In addition, b1 strategy is almost as precise as the gold standard HC2 which has a 1,241 diagnostic odds ratio. In this context, each scenario can be used under different circumstances depending on the objective pursued. Finally, Fig 6 shows how cohort results would have changed, from the original screening algorithm (Fig 2), if cytology-reflex or HPV-reflex strategies were considered for the initial screening.

**Table 2:** Estimated accuracy of cytology, Hybrid-2-Capture, and co-testing (two possible scenarios).

Classification Metric	Hybrid-2-Capture	Cytology	Co-Testing (b1)	Co-Testing (b2)
Sensitivity	0.99	0.64	0.64	0.99
Specificity	0.94	0.98	0.99	0.94
Positive Predictive Value	0.23	0.41	0.49	0.22
Negative Predictive Value	1.00	0.99	0.99	1.00
Positive Likelihood Ratio	956.27	62.18	74.47	899.38
Negative Likelihood Ratio	0.77	0.59	0.51	0.78
Diagnostic Odds Ratio	1,241	105	145	1,148

We evaluated the statistical parameters of both tests vs. follow-up (histological-High-Grade Squamous Intraepithelial Lesion (HSIL) as the primary end-point) in a) both tests independently and in b) combined (co-testing). Since the analysis is binomial, the following scenarios were proposed adding discordant co-testing to negative or positive binary values: b1) negative co-test = double-negative and discordant, and b2) positive co-test = double-positive and discordant. Each scenario can be used under different circumstances depending on the objective pursued.

## Discussion

### Main findings

Of all positive-Hybrid-2-Capture patients, 22.81% had an HSIL histological diagnosis which was timely and adequately treated; 37.43% showed viral persistence, and 39.77% viral clearance; these results differ from other published studies [4]. Most HPV infections are transient and viral clearance by the immune system occurs spontaneously without ever causing clinically relevant lesions [5]. Nevertheless, the extent to which viral infections are cleared remains a major unresolved issue [17]. Sichero et al. described that women with persistent high-risk HPV infections possess a 50-fold higher risk of developing cervical cancer when compared to negative-HPV women [17]. In our study, the multinomial logistic regression analysis showed that the HSIL odds ratio was 19.09 (P<0.001) for a positive-cytology. These results support a direct association with prognosis, as shown in Fig 5; in a cohort of Hybrid-2-Capture patients, negative-cytology showed a 0.50 probability of viral clearance (24 months), ASC-US P = 0.647 of viral persistence (24 months), and positive-cytology P = 0.647 of HSIL (Fig 5). Our decision to analyze ASC-US as an independent group from positive cytology enabled us to observe this pattern, suggesting that cytological changes observed in positive-Hybrid-2-Capture patients could affect prognosis with statistical certainty. The associations found have a progressive biological trend: negative-cytology/clearance, ASC-US/persistence, and positive-cytology/HSIL.

### Clinical implementation of a cervical cancer screening program

We have determined that the best scenarios included: a) standalone Hybrid-2-Capture (diagnostic odds ratio = 1,241) or b2) negative co-tests defined as only double negative cases (Table 2). Evidence from randomized clinical trials suggests that screening with cytology alone is less sensitive for detecting HSIL than screening with high-risk HPV testing alone. Cytology programs have been challenging to establish in low- and middle-income countries because quality assurance for accurate and reproducible results, requires better human and financial resources than other screening strategies [18]. Although screening with high-risk HPV testing alone or in combination with cytology has a higher HSIL detection rate, this strategy results in more diagnostic colposcopies [9]. Our study revealed similar findings. Although this might increase the cost of care in high-income countries, this is not the case in our country (average cost of colposcopy: USD$2).

### Future of cervical cancer

In Latin America, decades of cytology-based screening to detect precancerous cervical lesions have not made a significant impact in the reduction of cervical cancer incidence, morbidity, and mortality rates [17, 19, 20]. By 2030, the World Health Organization predicts a 27% rise in cervical cancer mortality in low- and middle-income countries compared with just a 1% increase in high-income countries, reflecting the social imbalance in healthcare systems between countries and within regions. The transition from cytology to molecular high-risk HPV testing for primary cervical cancer could be an opportunity to minimize the injustice for women in low- and middle-income countries. They possess a substantially increased likelihood of dying from cervical cancer simply because of their place of birth [21].

Over the last decade, diverse experiences with HPV testing have been conducted in Latin American countries, some as part of research studies, and others, more recently, to pilot the implementation of HPV testing in the public health system [19]. Argentina was the first country in Latin America to implement HPV DNA testing for primary screening within the public health system for all women aged 30 or older. Latin America is slowly shifting towards HPV testing for CC screening: “EStudio Multicentrico de TAMizaje y Triaje usando la prueba de PApilomavirus humano” (ESTAMPA-NCT01881659), which began on 01/06/2013. The aim is to study >50,000 women (30–64 years old) with HC2 or COBAS with IARC funding [20]. Hundreds of thousands of women will continue to die while the uneven distribution of healthcare resources continues. Eradication of cervical cancer could be near, but not until the global community stops ignoring the deaths of millions, and takes serious action towards enabling universal screening [22].

### Study limitations

To obtain the most reliable data, we have excluded patients who reported diagnosis of high-grade lesions in the last 20 years or had clinical findings of any HPV related condition before screening. This has led to significantly less enrollment in comparison to other similar studies. However, the positive-Hybrid-2-Capture rate was 8.45% which correlates with published evidence [11, 23]. This might present a different scenario observed while screening women in regions with deficiencies in healthcare, who lack previous studies. In terms of accessibility and quality of care, our study population could be likened to those in high-income countries, thus revealing the inequality within Argentina.

Our study was conducted mimicking the National Health Program that used hybrid capture. This technology was chosen for two reasons; on the one hand, for its safety aspects regarding self-sampling. On the other, in 2013, when our study began, hybrid capture was the only clinically validated HPV test available.

### Detection strategies

Currently, the two most used methods for HPV DNA detection are hybridization which uses a whole genome probe, and Polymerase-Chain-Reaction which uses L1 amplification. We chose Hybrid-2-Capture for several reasons; first and foremost, the L1 gene can be deleted in cervical cancers and lead to false-negative results. Tjalma *et al*. identified women who died of cervical cancer and multiple women with high-grade lesions who tested negative for L1 but positive for E6/E7. To prevent false negatives caused by gene deletions, the hybrid-capture method uses full genome RNA probes [24, 25]. Also, we chose Hybrid-2-Capture because of its simplicity and robustness, key features considering the lack of infrastructure in peripheral health centers; obtaining an excellent mean turnaround time of 19.31 days. And lastly, its versatility allows self-sampling, enabling women across many countries and age groups to carry out the test independently by following written instructions [26].

The shift from cytology-based screening to molecular HPV testing has required a steep learning curve for screening programs and the laboratories that support them [27]. Latin America lacks formal regulation for laboratories that perform HPV molecular tests, which means that any healthcare provider could use home-brew methods, non-validated methods, or even methods without an intra-laboratory learning curve. As for international accreditations, extremely few Latin American health centers possess them, not to mention that many peripheral health centers do not even have immunohistochemistry [28]. Ongoing validation/verification is also required annually to ensure that the assay is working consistently. The central longitudinal quality measures are internal quality assessment, external quality assessment schemes and endogenous controls (only for Polymerase-Chain-Reaction methods). These involve amplifying a housekeeping gene present in every human cell, such as β-globin, to detect the failure in extraction and inhibition of the Taq-polymerase. While endogenous controls can be helpful, they do not confirm that relevant cervical cells are present in the sample, just that human cells are [27].

For us, “a woman with cervical cancer is a sign of a fragile healthcare system”. We analyzed the performance of the three possible scenarios for cervical cancer screening in Latin America: cervical cytology (HPV-reflex), HPV testing (cytology-reflex), or HPV testing combined with cytology (co-testing). Their statistical analysis, as well as their performance and cost, determined that any of them could be used under different circumstances for the three patient profiles described for Latin America: i) women with favorable socioeconomic and cultural profiles who are most often diagnosed early; ii) women with a social vulnerability profile whose diagnosis is often too late, and iii) women with profound geographical barriers who will never know their cause of death [22]. In the face of these three realities, we thought about analyzing the three possible scenarios thoroughly, through statistical models, to convey the following concept: “HPV Test is a real tool against inequity in healthcare for Latin American women.”

### Conclusion

Even in this era of developmental technology, there are still several countries where women suffer and die from preventable diseases, such as cervical cancer. The five-year cumulative incidence rate of the high-grade lesion of our population was 1.73%, with a 8.45% hrHPV prevalence. The follow-up analysis of the study population at the end of the five years determined that 22.81% of the HC2-positive group had an HSIL histological diagnosis which was adequately treated; 37.43% had viral persistence, 39.77% had viral clearance. Our study provides critical, real-world evidence which could encourage low- and middle-income countries to incorporate co-testing as a cervical cancer screening tool. Multinomial logistic regression analysis supported a direct association with prognosis, i.e., negative-cytologyt/clearance, ASC-US/persistence, and positive-cytology/HSIL. If these findings are confirmed in more extensive population studies, cytology could be regarded as a prognostic factor on Hybrid-2-Capture positive women in a co-testing context. This evidence suggests that the introduction of screening via co-testing in low- and middle-income countries could diminish the burden of cervical cancer, acting as a tool against inequity in healthcare.

## Supporting information

S1 File Cohort database. (XLSX)
